# Supplementary material for: O-GlcNAcylation of nuclear proteins in the mouse liver exhibit daily oscillations that are influenced by meal timing
Source: PLoS Biol. 2025 Sep 25;23(9):e3003400. doi: 10.1371/journal.pbio.3003400 (PMC12500093; doi:10.1371/journal.pbio.3003400)
Supplement: S1 Table — (DOCX) [file pbio.3003400.s010.docx]

**S1 Table. Comparison between this study and published liver phosphoproteome datasets.**

| Reference | Number of phospho-peptides | Number of phospho-proteins | Number of oscillating phospho-peptides (Percentage) | Number of oscillating phospho-proteins (Percentage) | Circadian kinases | Normalize to protein level | Label method | TRF | LD vs DD | Cell fraction | Time points X days | Replicate number (total sample number) |
| --- | --- | --- | --- | --- | --- | --- | --- | --- | --- | --- | --- | --- |
| This study | 19593 | 3272 | 1054 (5.38%) | 579 (17.70%) | AKT, S6K, CDK, VRK | Yes | TMT | Yes (3 weeks) | LD | Nucleus | 6 time points X 1 day | 3 (18) |
| Robles et al.[1] | 7896 | 2672 | 2123 (26.89%) | 1088 (40.71%) | ERK, MEK, RSK, JNK, AKT, mTOR, p70S6K Kinase,  CK1δ | No | No | No | DD | Whole cell | 8 time points X 2 days | 4 (64) |
| Huang et al.[2] | NA | 2639 | NA | 1668 (63.21%) | NA | No | No | Yes (1 week) | LD | Whole cell | 6 time points X 2 day | 4 (48) |
| Wang et al.[3] | 9465 | 1657 | NA | 89 (5.37%) | NA | No | No | No | LD | Whole cell | 8 time points X 2 days | 1 (Pooled 3 mice) (16) |
| Wang et al.[4] | 1448 | NA | 154 (10.64%) | 113 | GSK3α, GSK3β,CK1α, CK1δ, CDK1, CDK4, CDK6 | No | SILAC | Yes (4 days) | LD | Nucleus | 8 time points X 2 days | 1 (16) |

**References**

1. Robles MS, Humphrey SJ, Mann M. Phosphorylation Is a Central Mechanism for Circadian Control of Metabolism and Physiology. Cell Metabolism. 2017;25: 118–127. doi:10.1016/j.cmet.2016.10.004

2. Huang R, Chen J, Zhou M, Xin H, Lam SM, Jiang X, et al. Multi-omics profiling reveals rhythmic liver function shaped by meal timing. Nat Commun. 2023;14: 6086. doi:10.1038/s41467-023-41759-9

3. Wang Y, Song L, Liu M, Ge R, Zhou Q, Liu W, et al. A proteomics landscape of circadian clock in mouse liver. Nat Commun. 2018;9: 1553. doi:10.1038/s41467-018-03898-2

4. Wang J, Mauvoisin D, Martin E, Atger F, Galindo AN, Dayon L, et al. Nuclear Proteomics Uncovers Diurnal Regulatory Landscapes in Mouse Liver. Cell Metabolism. 2017;25: 102–117. doi:10.1016/j.cmet.2016.10.003
