# Supplementary material for: O-GlcNAcylation of nuclear proteins in the mouse liver exhibit daily oscillations that are influenced by meal timing
Source: PLoS Biol. 2025 Sep 25;23(9):e3003400. doi: 10.1371/journal.pbio.3003400 (PMC12500093; doi:10.1371/journal.pbio.3003400)
Supplement: S2 Table — (DOCX) [file pbio.3003400.s011.docx]

**S2 Table. Known functions of rhythmic phosphorylation sites that are identified in protein complexes.**

| Gene name | Uniprot ID | Phosphosite | Kinase | Function | Reference |
| --- | --- | --- | --- | --- | --- |
| *Hdac1* | O09106 | S421, S423 | CK2 | Phosphorylation of S421 and S423 promotes the deacetylase activity and complex formation of HDAC1 | [1] |
| *Clock* | O08785 | T461 | CDK5 | T461 phosphorylation reduces stability and promotes nuclear translocation of CLOCK | [2] |
|  |  | S431 | NA | Phosphorylation of S431 primes the phosphorylation of S427 | [3] |
|  |  | S427 | GSK3β | Phosphorylation of S427 leads to CLOCK degradation | [3] |
|  |  | S845 | AKT | Phosphorylation of S845 inhibits its nuclear translocation and affects circadian gene expression | [4] |
| *Rbl2* | Q64700 | S410, S659 | NA | Phosphorylation of S410 and S659 (S413 and S662 in human homolog) promotes the dissociation between RBL2 and E2F4, and relieves repression of E2F4 target gene expression. | [5] |
| *Sfpq* | Q8VIJ6 | T679 | GSK3 | Phosphorylation of T679 (T687 in human homolog) increases the interaction between SFPQ and TRAP150, which inhibits the binding of SFPQ pre-mRNA and thus affects its RNA splicing activity. | [6] |

**Supporting Information References**

1. Pflum MK, Tong JK, Lane WS, Schreiber SL. Histone deacetylase 1 phosphorylation promotes enzymatic activity and complex formation. J Biol Chem. 2001;276: 47733–47741. doi:10.1074/jbc.M105590200

2. Kwak Y, Jeong J, Lee S, Park Y-U, Lee S-A, Han D-H, et al. Cyclin-dependent kinase 5 (Cdk5) regulates the function of CLOCK protein by direct phosphorylation. J Biol Chem. 2013;288: 36878–36889. doi:10.1074/jbc.M113.494856

3. Spengler ML, Kuropatwinski KK, Schumer M, Antoch MP. A serine cluster mediates BMAL1-dependent CLOCK phosphorylation and degradation. Cell Cycle. 2009;8: 4138–4146. doi:10.4161/cc.8.24.10273

4. Luciano AK, Zhou W, Santana JM, Kyriakides C, Velazquez H, Sessa WC. CLOCK phosphorylation by AKT regulates its nuclear accumulation and circadian gene expression in peripheral tissues. J Biol Chem. 2018;293: 9126–9136. doi:10.1074/jbc.RA117.000773

5. Farkas T, Hansen K, Holm K, Lukas J, Bartek J. Distinct phosphorylation events regulate p130- and p107-mediated repression of E2F-4. J Biol Chem. 2002;277: 26741–26752. doi:10.1074/jbc.M200381200

6. Heyd F, Lynch KW. Phosphorylation-dependent regulation of PSF by GSK3 controls CD45 alternative splicing. Mol Cell. 2010;40: 126–137. doi:10.1016/j.molcel.2010.09.013
