# Supplementary material for: O-GlcNAcylation of nuclear proteins in the mouse liver exhibit daily oscillations that are influenced by meal timing
Source: PLoS Biol. 2025 Sep 25;23(9):e3003400. doi: 10.1371/journal.pbio.3003400 (PMC12500093; doi:10.1371/journal.pbio.3003400)
Supplement: S3 Table — (DOCX) [file pbio.3003400.s012.docx]

**S3 Table. The domains containing or near rhythmic phosphosites with unknown function (only phosphosites identified in protein complexes are included).**

| Gene name | Uniprot ID | Phosphosite | Nearby domains* (amino acid number of the domains) | Distance from nearby domain |
| --- | --- | --- | --- | --- |
| *Ptbp2* | Q91Z31 | S308 | RNA recognition motif (aa309-434) | -1 |
| *Hnrnpm* | Q9D0E1 | S636 | RNA recognition motif (aa654-722) | -18 |
| *Rbl2* | Q64700 | Y632 | RB A (aa414-606) | +26 |
| *Gatad2b* | Q8VHR5 | S123 | p66 CC (158-201) | -35 |
| *Mbd3* | Q9Z2D8 | Y83 | MBDa (79-148) | 0 |
| *Sin3a* | Q60520 | S939 | Sin3a C (887-1187) | 0 |
| *Smarca4* | Q3TKT4 | S1388 | SnAC (1289-1356) | +32 |
| *Med7* | Q9CZB6 | S195 | Med7 (7-164) | +31 |
| *Clock* | O08785 | S91 | HLH (35-85) | +6 |
|  |  | S94 | HLH (35-85) | +9 |
|  |  | S403 | PAS 11 (273-380) | +23 |
|  |  | S406 | PAS 11 (273-380) | +26 |
|  |  | S408 | PAS 11 (273-380) | +28 |
| *Ncl* | P09405 | S605 | RRM1 (574-641) | 0 |
|  |  | S462 | RRM1 (395-460) | +2 |
|  |  | T307 | RRM1 (309-377) | -2 |
|  |  | S563 | RRM1 (489-555) and RRM1 (571-638) | +8 and -8 |
| *Smc1a* | Q9CU62 | S649 | SMC hinge SMC N (512-629) | 0 |
