# Supplementary figures and images for: O-GlcNAcylation of nuclear proteins in the mouse liver exhibit daily oscillations that are influenced by meal timing
Source: PLoS Biol. 2025 Sep 25;23(9):e3003400. doi: 10.1371/journal.pbio.3003400 (PMC12500093; doi:10.1371/journal.pbio.3003400)

Fig. 1A

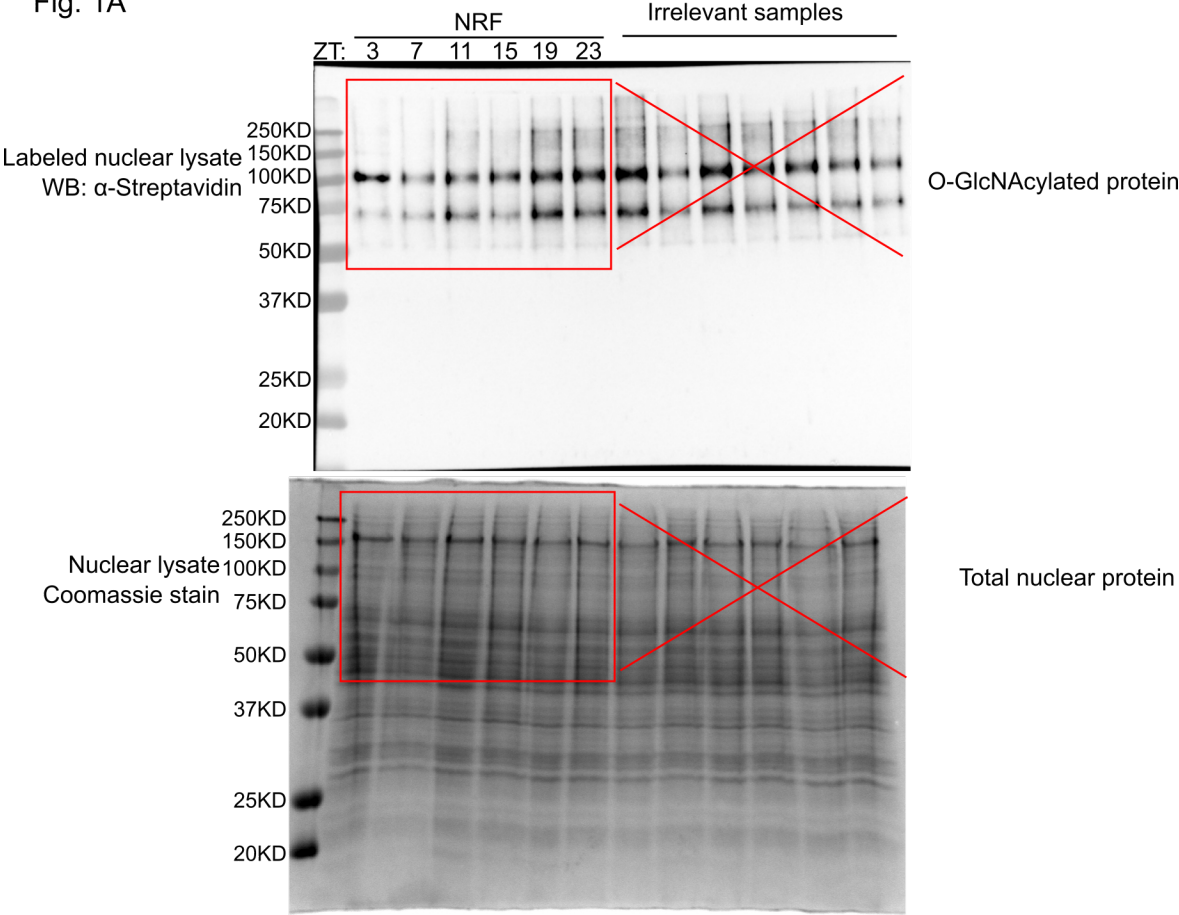

Fig. 5C

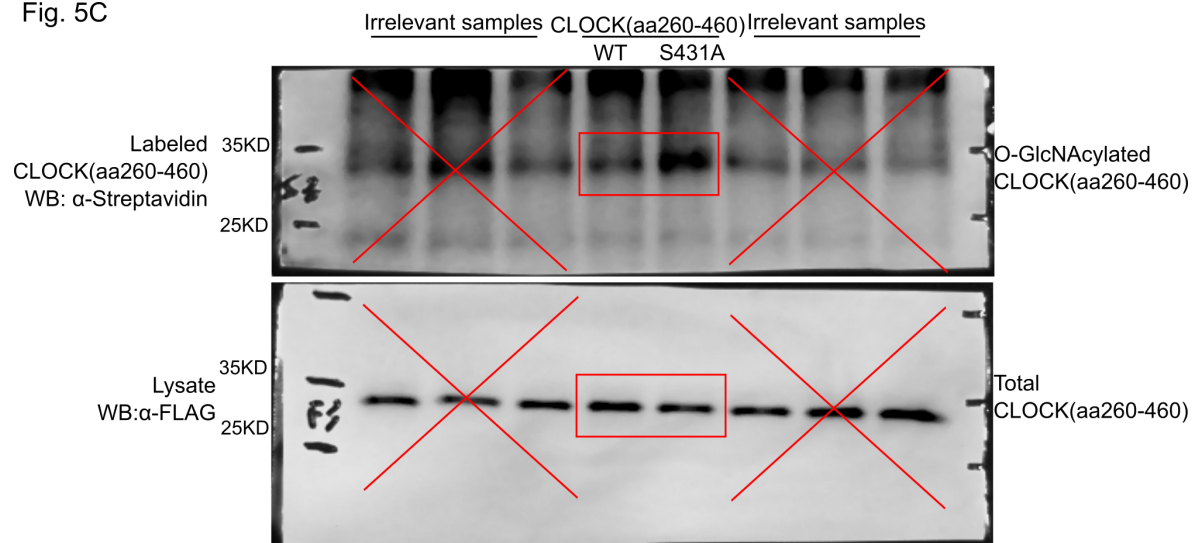

Fig. 5E

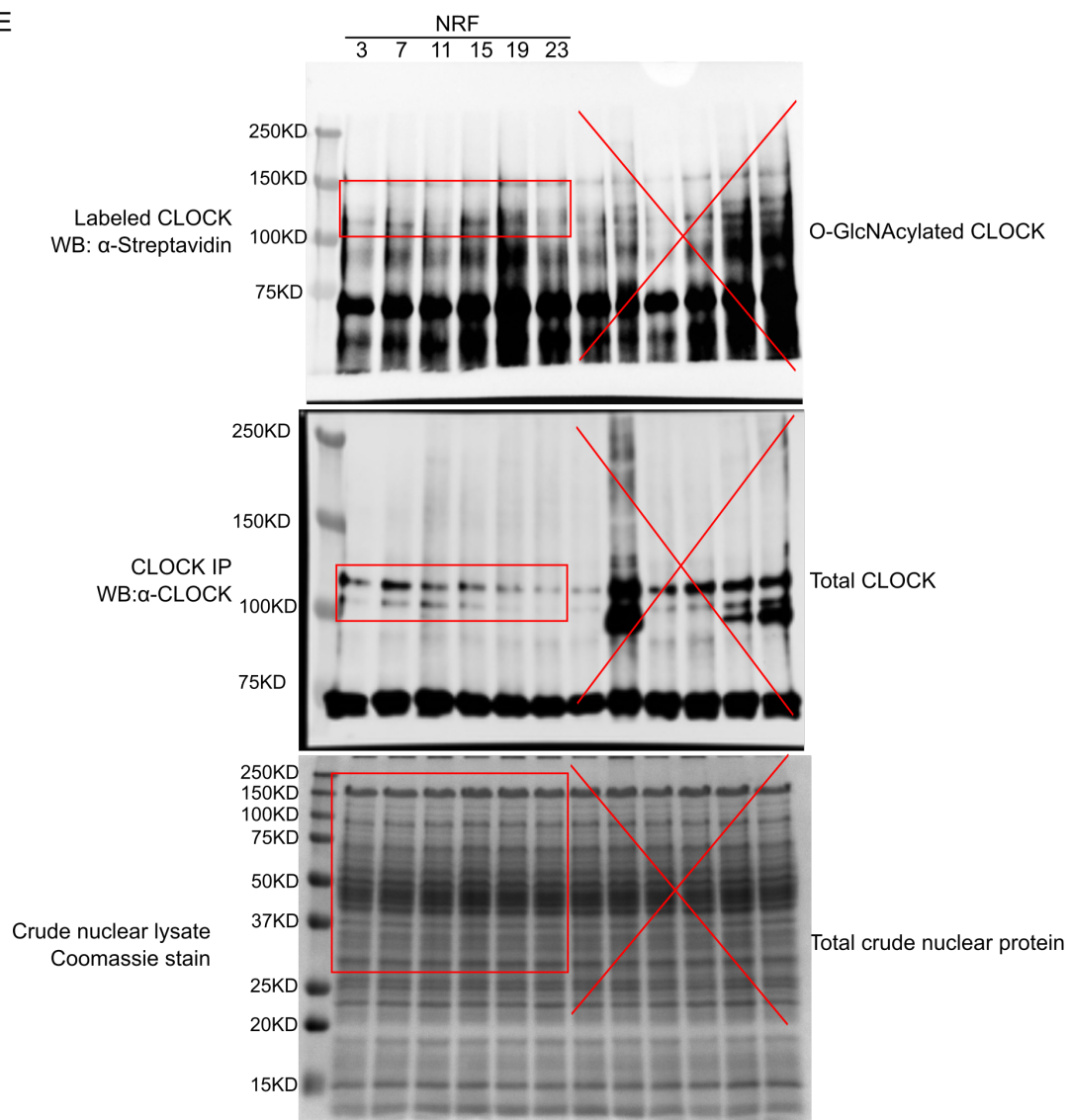

Fig. 6B

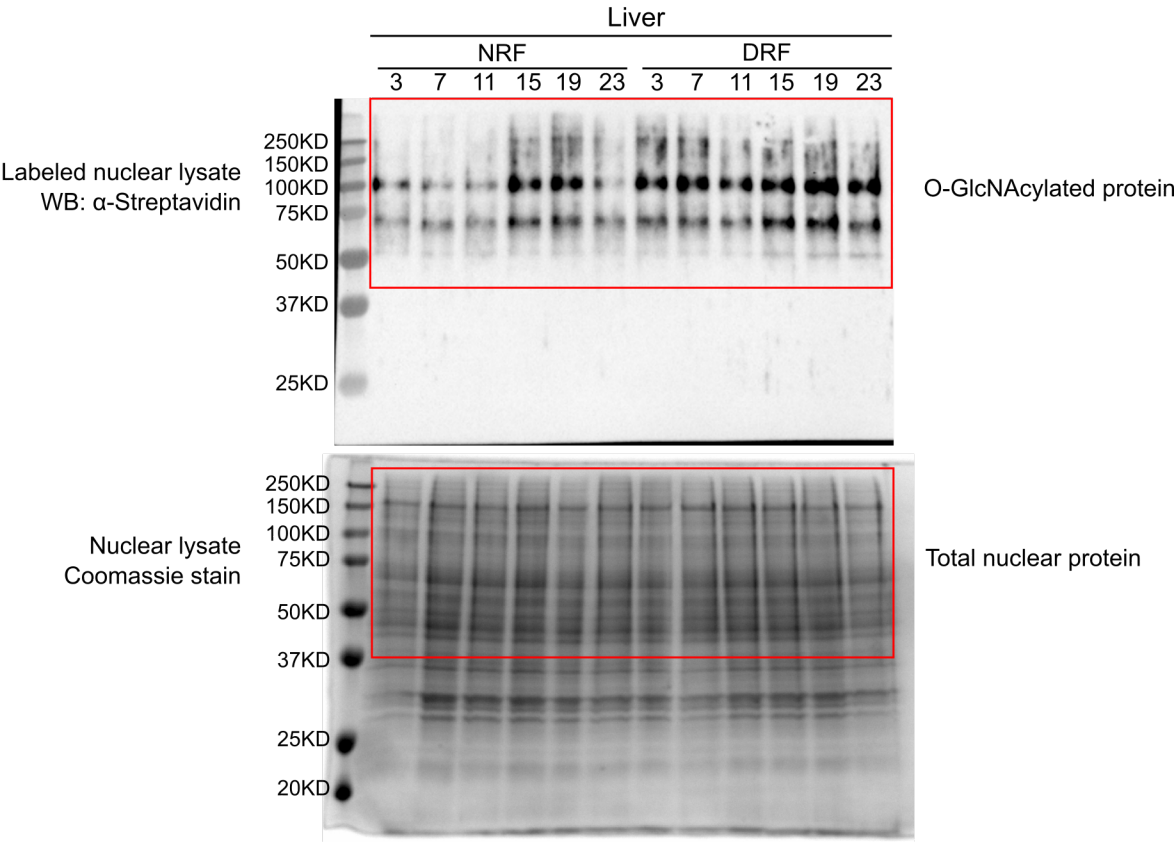

Fig. S1

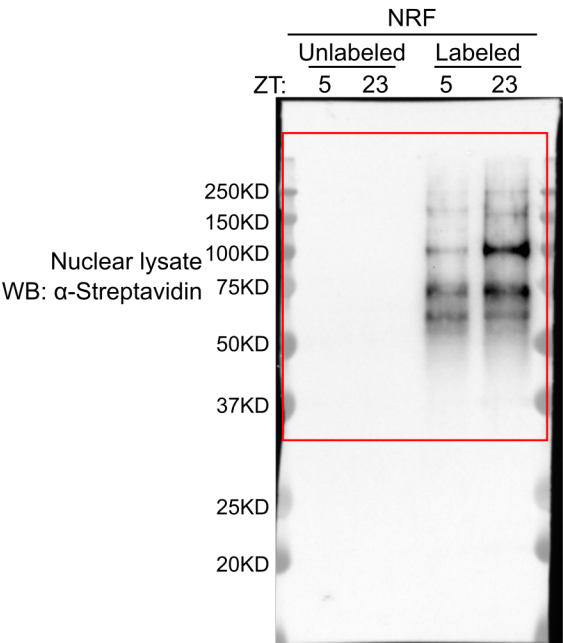

Fig. S8A

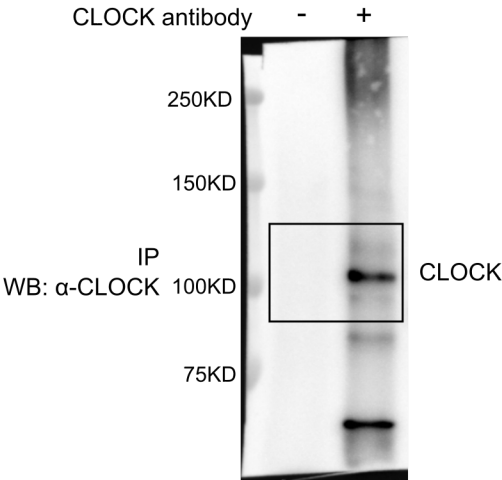

Fig. S8B

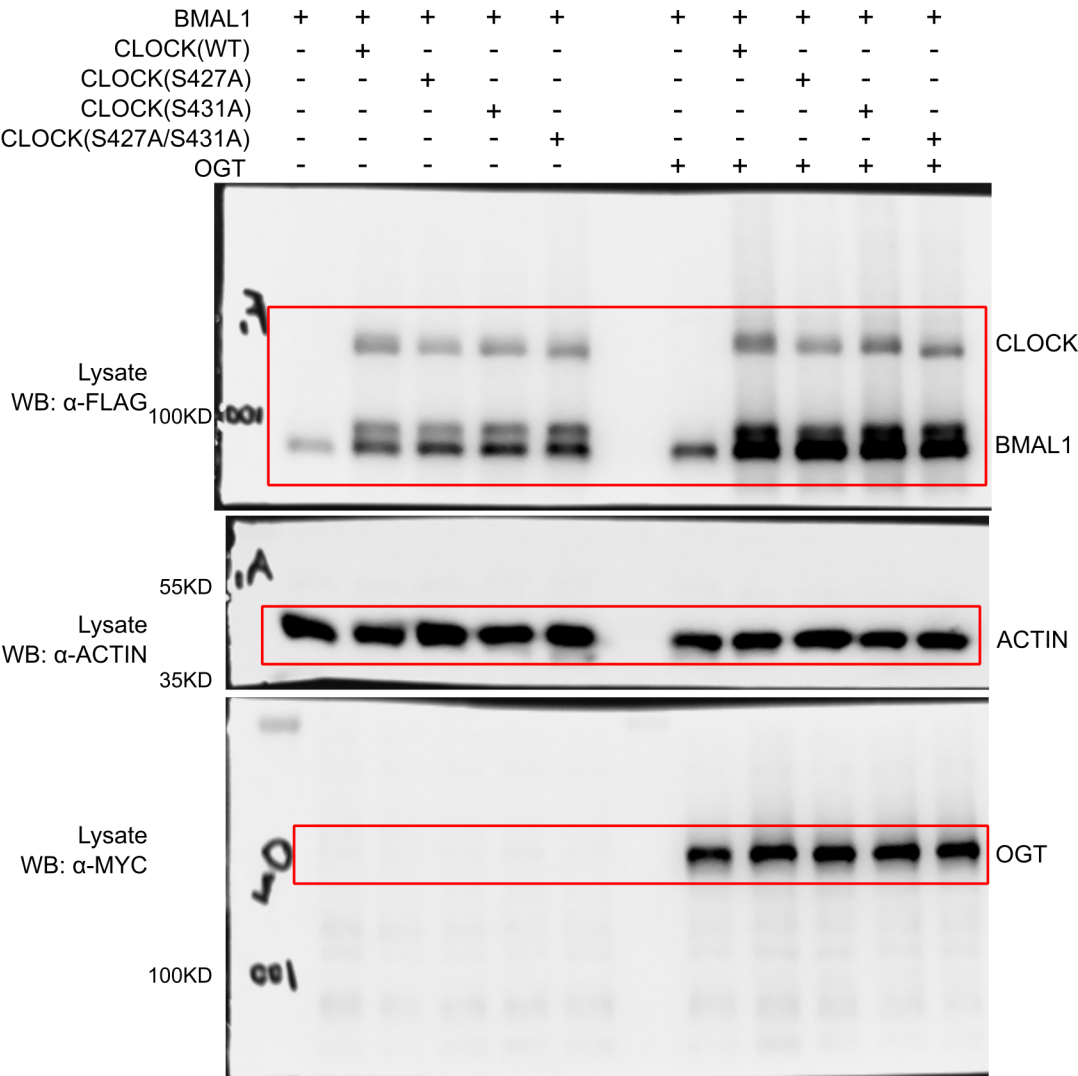

Supplement: S1 Raw Images — (PDF) [file pbio.3003400.s024.pdf]
