# Supplementary material for: O-GlcNAcylation of nuclear proteins in the mouse liver exhibit daily oscillations that are influenced by meal timing
Source: PLoS Biol. 2025 Sep 25;23(9):e3003400. doi: 10.1371/journal.pbio.3003400 (PMC12500093; doi:10.1371/journal.pbio.3003400)
Supplement: Attachment — Submitted filename: Response_250523.pdf [file pbio.3003400.s027.pdf]

We thank the editor and reviewers for their constructive comments and suggestions and for the opportunity to submit a revised manuscript. As suggested, we performed additional experiments necessary to support the claims we made in the manuscript. Below, we provide point-by-point responses that address the reviewers' concerns. Our responses are in black regular font, and the reviewers' comments are in *red italics font*. For the convenience of the reviewers, we are submitting a "marked-up" copy of the manuscript with key changes highlighted by "track-changes".

REVIEWS:

Reviewer #1: In their manuscript "Mealtime alters daily rhythm in nuclear O-GlcNAcome to regulate hepatic gene expression," Liu et al. provide evidence that nuclear protein O-GlcNAcylation in the mouse liver undergoes circadian oscillation that is disrupted by unnatural daytime feeding. Using O-GlcNAcomic and phosphoproteomic data, the authors suggest that the crosstalk between O-GlcNAcylation and phosphorylation plays a crucial role in shaping circadian hepatic gene expression and protein function.
The nuclear characterization of the O-GlcNAcome and phosphoproteome in the liver around the clock can serve as a valuable resource, however as detailed below these analyses suffer from some major drawbacks.
The main claim of the authors, as indicated in the title "Mealtime alters daily rhythm in nuclear O-GlcNAcome to regulate hepatic gene expression," is not supported by the data, and therefore the title is misleading. Overall, the study is primarily based on correlational data. This limitation makes it difficult to draw any definitive conclusions about the impact of circadian nuclear protein O-GlcNAcylation on hepatic gene expression as well as on other conclusions throughout the study.
Major concerns:
* *There are several major issues with the statistical analyses in the study: RAIN is a very permissive analysis for detecting rhythms. It would be good to complement this analysis with JTK cycle or other relevant algorithms and the false discovery rate (FDR) should be included. The statistical analysis is flawed as the authors rely on p-values instead of q-values that correct for FDR and are widely accepted in the field (see guidelines paper PMID: 29098954). Without proper statistical analysis, the value of the study as a resource is diminished.*

We thank the reviewer for the suggestion to improve our study. We have added FDR for our RAIN analysis (Fig. 2C and 3C). In addition, we analyzed the O-GlcNAc proteomics and TMT phosphoproteomics data with JTK cycle (Hughes et al., 2010 JBR). To further increase transparency, we have now reported the number of rhythmically modified proteins/peptides according to different RAIN and JTK q value cut-offs (0.1, 0.2, 0.3 and 0.4). Following previous omic-scale analyses, we used RAIN q<0.2 as cut-off for further analysis (Malik et al., 2023, bioRxiv; Malik et al., 2024, J. Proteome Res). To further confirm time of day variation in our O-GlcNAc proteomic and phosphoproteomic dataset, we performed PLS-DA analysis (Tennessen et al., 2014, Methods). Both protein modifications exhibit overall rhythmicity (Fig 2A and B, Fig 3A and B).

* *As the author state "The current technology to identify and quantify O-GlcNAc sites on*

proteins is not yet mature based on consultation with experts in the field, as the labile glycosidic bond of O-GlcNAcylation posed great challenge for mass spectrometry analysis," together with the batch effect issues, raise further concerns regarding the validity of the data.

After much optimization, we have now included TMT quantitative profiling of site-specific O-GlcNAcylation of liver proteins extracted from mice at two time-points, ZT11 (day) and ZT23 (night) (Lines 220-244). These mice were subjected to night-restricted feeding (NRF), same as mice used for label-free global O-GlcNAcylation analysis. Based on daily patterns of global protein O-GlcNAcylation in Western blot (Fig 1A, 1B) and label-free global O-GlcNAc proteomics (Fig 2D, 2E), which were performed at 6 time-points over the day-night cycle, we chose to analyze nuclear proteins from mouse liver collected at ZT11 and ZT23 because they represent a day and a night time-point that showed clear differences. In total, we detected 790 high-confidence O-GlcNAc peptides, belonging to 346 proteins. Over 40% of O-GlcNAcylated proteins identified by TMT site-specific analysis were also identified in our label-free global O-GlcNAc proteomics (Fig 2F). Multivariate PLS-DA analysis showed that O-GlcNAcylation sites quantified by TMT-MS exhibits day-night differences (Fig 2G). 125 O-GlcNAc sites on 101 proteins showed day-night O-GlcNAcylation differences (>20%-fold changes) (Fig 2H). Consistent with O-GlcNAcylation determined by Western blot (Fig 1A-B) and label-free global analysis (Fig 2D-E), over 60% of the 152 O-GlcNAc peptides quantified by TMT-MS display higher O-GlcNAcylation at ZT23 (nighttime) (Fig 2H and I). Notably, we also found sites that exhibit lower O-GlcNAcylation at daytime. We reasoned these sites potentially belong to less abundant nuclear proteins that are not representative in the profile shown in Western blot results (Fig 1A-B), highlighting the value of site-specific analysis, as suggested by the reviewer. Our new results provide further support for temporal regulation of site-specific O-GlcNAcylation over the day-night cycle.

To gain insight into whether site-specific O-GlcNAcylation is responsive to feeding time, we also performed TMT site-specific O-GlcNAc proteomics for nuclear proteins extracted from mice subjected to day-restricted feeding (DRF) and compared to that of NRF nice (Fig. 6D-F). Consistent with global nuclear protein O-GlcNAcylation detected by Western blot (Fig. 6B-C), DRF leads to reduced number of peptides (from 161 to 124) with day-night differences in O-GlcNAcylation, as compared with that of NRF (Fig 6D). Notably, O-GlcNAcylation level in the majority of peptides is responsive to altered feeding time (Fig 6E), with some peptides showing inverted day-night patterns between NRF and DRF. Similar to above, site-specific O-GlcNAcylation provides additional insights into the regulation of temporal O-GlcNAcylation. Our data support our conclusion that timing of food intake modulates daily O-GlcNAcylation rhythm in mouse liver (Lines 413-427).

* *In the section titled "Daily O-GlcNAcomics identifies rhythmically O-GlcNAcylated nuclear proteins," the authors report proteomics analyses of only 2 animals (n = 2), which corresponds to low statistical strengthes (see guidelines paper PMID: 29098954).*

We have reanalyzed our O-GlcNAc proteomics dataset with 3 biological replicates (Fig 2A-E), which did not show much batch effects.

As listed below there are several overstatements throughout the study that are not supported by

data:

**   In the section titled "Crosstalk between O-GlcNAcylation and phosphorylation (G-P) regulates hepatic protein function," the authors utilize correlational evidence. Without directly testing the functional interactions through loss-of-function experiments, the correlation shown here does not definitively establish the "crosstalk" or the regulation of hepatic protein function.*

As suggested by the reviewer, we have now included functional characterization of G-P crosstalk in the CLOCK protein as a case study (Lines 386-404). We chose CLOCK because of its relevance to circadian rhythms. Since phosphorylation at S427 triggers degradation of CLOCK (Spengler et al., 2009, Cell Cycle), we hypothesized that O-GlcNAcylation at S431 residue can regulate CLOCK stability and transcriptional activity through inhibiting stepwise phosphorylation at S431 and S427 (Fig 5B). In the previous version of the manuscript and also shown in the revised version (Fig 5E to G), we presented the daily rhythms of CLOCK O-GlcNAcylation, pCLOCK(S427), and overall CLOCK protein level. Our results showed correlative evidence supporting our hypothesis that CLOCK O-GlcNAcylation could inhibit pCLOCK(S427) (Fig 5E and F). To further test this hypothesis as suggested by the reviewer, we constructed CLOCK(S427A), CLOCK(S431A), and CLOCK(S427A/S431A) mutants and expressed them in HEK293T cells. We first confirmed that CLOCK S431 is a O-GlcNAc site by showing that O-GlcNAcylation level of CLOCK(aa260-460)-S431A is lower than CLOCK(aa260-460)-WT (Fig 5C and D). For these experiments, we used a shorter CLOCK construct to minimize the effects caused by other modified residues on CLOCK.

We then performed *period-luciferase* reporter assay to investigate the transcriptional activity of CLOCK variants as compared to WT in the presence and absence of OGT (*period* is a direct target of CLOCK transcriptional activity). In published literature (e.g., Kwon et al., 2006 MCB; Lee et al., 2008 MCB; Spengler et al., 2009 Cell Cycle; Stratmann et al., 2012 Mol Cell), lower CLOCK transcriptional activity is tightly linked to higher stability. This may seem counter-intuitive, but it has been established that qualitative changes in highly unstable CLOCK promote its transcriptional activity (also seen in many other transcription factors (reviewed in Thomas and Tyers, 2000 Curr Biol; Tansey, 2001 G&D). As expected, we observed CLOCK(WT) increases *per-luciferase* signal and this signal is significantly reduced when CLOCK(WT) is coexpressed with OGT. This makes sense given Li et al. (2013) previously showed that CLOCK O-GlcNAcylation increases its stability. We then tested the single mutants (S427A and S431A) and double mutant (S427/431A) and observed that they all exhibited lower transcriptional activity when compared to CLOCK(WT), and coexpression of OGT did not impact *per-luc* activity (Fig 5H). Taken together, these data demonstrate the crosstalk between O-GlcNAcylation and phosphorylation regulates protein function and support the model that CLOCK S427 and S431 regulates as a unit to impact CLOCK activity.

**   The section titled "Daily G-P crosstalk on CLOCK and RBL2" as well contains only correlative evidence. The graph of RBL2 (Fig 5H) looks particularly unconvincing as evidence of G-P crosstalk in RBL2 protein regulation. The authors, again, provide no functional tests that directly assess these particular O-GlcNAcylations/phosphorylations. Therefore, all the conclusions drawn are merely speculative. Additional gain- or loss-of-function experiments are necessary to conclusively demonstrate causal relationships.*

We have performed functional characterization of the G-P crosstalk on CLOCK as mentioned above (Fig 5). We deleted the discussion on RBL2 because we did not perform functional analysis on RBL2 and did not want to overstate our conclusions, as suggested by the reviewer.

*   *In the section titled "O-GlcNAcylation of nuclear proteins in liver regulates daily hepatic transcriptome," the authors claim to assess gene expression without indicating the use of standard gene expression measurement techniques such as RNA-seq. Instead, the conclusions primarily rely on correlations between O-GlcNAcylation and phosphorylation patterns.*

We apologize for the confusion. We decided to take this section out so as not to overstate our conclusion. We agree with the reviewer that simply correlating O-GlcNAcylation and phosphorylation rhythms of transcription factors with transcription rhythms could not provide much insight into the function of these PTMs without additional functional analysis, such as the experiments we performed to characterize CLOCK G-P interplay (as described above). Indeed, many other factors are involved in the regulation of gene expression, such as chromatin remodeling, histone modification, RNA processing, etc.

*   *The authors "report that unnatural day time-restricted feeding (DRF) dampens O-GlcNAcylation rhythm, suggesting the disruption in liver transcriptomic rhythm previously observed in DRF condition could be mediated by dysregulation of daily protein O-GlcNAcylation rhythm." However, since DRF inverts the rhythmic transcriptome in the liver (e.g., PMID: 34059820), and dampening of the O-GlcNAcylation rhythm does not correspond to this 12 h shift, it actually argues against the authors' claim. This point merits discussion.*

We understand the reviewer's concern and have edited our Abstract, especially given the addition of our new site-specific O-GlcNAcylation data (Lines 43-50). We now state "we reported that day-restricted feeding (DRF) in the nocturnal mouse significantly altered O-GlcNAcylation rhythm. Whereas global O-GlcNAcylation analysis indicated dampening of global O-GlcNAcylation rhythm in mice fed under DRF, site-specific analysis revealed differential responses of O-GlcNAc sites when timing of food intake was altered. Notably, substantial O-GlcNAcylation exhibited an inverted day-night profile when mice were fed with DRF. This suggests the dysregulation of daily nuclear protein O-GlcNAcylation rhythm may contribute to the disruption in liver transcriptomic rhythm previously observed in DRF condition".

In our new site-specific analysis, we saw that some proteins actually exhibited inverted day-night O-GlcNAcylation patterns when mice were fed DRF instead of NRF (Fig 6E). This pattern was not obvious in the global O-GlcNAcylation analysis. It is possible that these proteins contribute to the previously observed DRF-induced phase shift of rhythmic genes (Damiola et al., 2000 G&D; Asher and Sassone-Corsi, 2015 Cell; Longo and Panda, 2016 Cell Metab; Manella et al., 2021 Nat Metab; Xin et al., 2021 iScience), but future functional experiments will be necessary to test this hypothesis. This is now discussed in the results and discussion of the revised manuscript (Lines 413-427, 502-511).

Finally, we also expanded our discussion (Lines 512-529) to provide a more balanced view. We clarify that in addition to nuclear O-GlcNAc proteome presented in this study, other mechanisms

are also involved in regulating the rhythms of daily hepatic transcriptome in addition to O-GlcNAcylation of nuclear proteins. These additional mechanisms could include O-GlcNAcylation of proteins in other subcellular compartments. Briefly, O-GlcNAcylation of cytoplasmic proteins, e.g. Raptor (Xu et al., 2023 Mol Cell), has been shown to regulate gene expression. Other nutrient-sensitive signaling pathways or PTMs can also interact with nuclear and cytoplasmic protein O-GlcNAcylation. Our metabolomics data also showed that daily profiles of a number of metabolites were altered in response to DRF (Fig S9). For example, altered S-adenosylmethionine (SAM) can regulate gene expression through influencing histone methylation (Wei et al., 2021 Cancer Res; Katada et al., 2010 Nat Struct Mol Biol; Aguilar-Arnal et al., 2015 Nat Struct Mol Biol). In summary, integration of nuclear and cytoplasmic protein O-GlcNAcylation, and other nutrient-sensitive pathways is required to better understand how meal timing regulates the daily transcriptome. Our goal for this study is to contribute to that understanding.

* *The section titled "Food intake at unnatural day feeding time in mice disrupts protein O-GlcNAcylation rhythm" provides limited data and falls short of linking the observed changes in O-GlcNAcylation to specific impacts on gene expression. As Fig 7D shows the daily pattern of UDP-GlcNAc remains rhythmic under both feeding conditions, it is unclear how the authors conclude that altered UDP-GlcNAc can possibly contribute to the losss of rhytmic O-GlcNAcylation in DRF mice liver. To demonstrate causal relationships, the authors should consider a direct manipulation of UDP-GlcNAc concentration in vivo e.g., by utilizing a GFAT inhibitor.*

We apologize for the confusion. In Fig 7D (now Fig 6G), UDP-GlcNAc is rhythmic under NRF condition (RAIN p=0.014) and arrhythmic under DRF conditions (RAIN p = 0.16), which correlates with dampened O-GlcNAcylation rhythm (Fig 6C). We have highlighted the RAIN p value in the graph.

Minor concerns:
* *In the section titled "Hepatic nuclear protein O-GlcNAcylation exhibit robust daily oscillation," the authors claim that "the timing of food intake occurred earlier than the rise of UDP-GlcNAc level (feeding: ZT12-24; UDP-GlcNAc: ZT 3-7) (Fig 1C)." However, with a broad feeding time window of 12 hours, it is difficult to determine which event precedes the other. The claim should be revised.*

We understand the reviewer's suggestion but we felt that the way we describe our data is the most factual. Nonetheless, we now elaborated on this sentence and states that "Although mice were allowed to feed from ZT12 to 24, the majority of feeding events are expected to occur earlier in the dark period (Koronowsky et al., 2019 Cell; Greenwell et al., 2019 Cell Rep)". (Lines 175-176).

* *The authors write at the end of the first section in the results: "Our data suggest that feeding-fasting cycles could drive the oscillation of UDP-GlcNAc, which results in the oscillation of protein O- GlcNAcylation". As no manipulation of feeding was done at this stage (mice were under natural night-time feeding), the conclusion is limited to rhythmicity, and whether this is feeding-determined remains unclear at this stage of the manuscript. The text should be revised.*

This sentence is mainly used to set up the rest of the manuscript, specifically to provide a rationale for studying protein O-GlcNAcylation. We have added one sentence to clearly state "The impact of meal timing on UDP-GlcNAc and O-GlcNAcylation rhythms will be evaluated in later sections of the study" (Lines 178-179).

* *Figure 3D, the authors can improve the panels. "The gray lines indicate the oscillation of each phosphopeptide, while the orange lines indicate the average phosphorylation pattern of each protein subunit." However, there is more than one protein in a panel, and it is impossible to attribute the gray lines to one or the other protein. Additionally, for TFIID, there are no gray traces?*

We apologize for the confusion. The aim of Fig 3D (now Fig 3F in the revision) is to show whether the phosphorylation of each subunit in the same protein complexes co-occur at the same time. We felt that plotting the phosphorylation of the subunits together will be more effective than plotting phosphorylation status of individual subunits separately. When only one peptide of individual protein was rhythmically phosphorylated (Per2-Sfpq-Sin3A complex panel), we used orange lines to represent phosphorylation status of individual peptides, hence there are no gray lines in that specific panel. We now clarified that in the figure legend (Lines 1311-1314). When multiple peptides were rhythmically phosphorylated within one single protein, we used gray lines to show phosphorylation status of individual peptides for each subunit. We did not want to further complicate the figure panels by introducing different colors to peptides of each subunit. Mostly, we did not feel that there is a need given our goal is to illustrate there is various degree of time correlation between phosphopeptides within protein complexes. Detailed phosphorylation data are also presented further discovery in S6 file and S9 file.

* *The text refers to Fig 4I, which does not seem to exist.*

We edited the text according to this comment.

* *The authors report on CLOCK, but what about BMAL1" BMAl1 was previously reported to be modified as well (Reference 32 in the manuscript shows a more pronounced rhythm in BMAL1 modification compared to CLOCK).*

Our O-GlcNAc proteomics did not detect BMAL1 O-GlcNAcylation in mouse liver. This could be because CLOCK is preferentially O-GlcNAcylated in CLOCK-BMAL1 complex in mouse liver, as compared with U2OS cells used in the original Reference 32 (Li et al., 2013, Cell Metab.).

* *The IP blot in Fig 5C can be improved. It lacks a control for non-specific binding (e.g., empty beads or IgG). In its current form, the specificity of the pulled-down protein CLOCK is questionable.*

As suggested by the reviewer, we performed the experiment that validated the specificity of CLOCK immunoprecipitation. As shown in Fig S8A, compared with empty beads, CLOCK was detected in immunoprecipitates of α-CLOCK bound beads.

Reviewer #2: In this work, Liu et al. explored the multi-omics landscape of mouse livers under night time-restricted feeding and provided a remarkable insight on the crosstalk between O-GlcNAcylation and phosphorylation in the context of meal timing. They generated a valuable resource and raised a plethora of interesting hypotheses for future research. In particular, they nailed down a new interaction between a phospho-degron and O-GlcNAcylation on CLOCK, an essential circadian clock protein. The work is technically vigorous and logically sound.

Major comments:
(1) The conclusion that "DRF dampens O-GlcNAcylation rhythm, suggesting the disruption in liver transcriptomic rhythm previously observed in DRF condition" requires additional evidence and sufficient control of the assays. Firstly, it is generally agreed that DRF reverses the liver clock as well as the hepatic rhythmic transcriptome (Refs from the labs of Schibler, Sassone-Corsi, Panda, Asher, Lazar etc.). The statement on transcriptome needs to be balanced.

We understand the reviewer's concern and have edited our Abstract, especially given the addition of our new site-specific O-GlcNAcylation data (Lines 43-50). We now state "we reported that day-restricted feeding (DRF) in the nocturnal mouse significantly altered O-GlcNAcylation rhythm. Whereas global O-GlcNAcylation analysis indicated dampening of global O-GlcNAcylation rhythm in mice fed under DRF, site-specific analysis revealed differential responses of O-GlcNAc sites when timing of food intake was altered. Notably, substantial O-GlcNAcylation exhibited an inverted day-night profile when mice were fed with DRF. This suggests the dysregulation of daily nuclear protein O-GlcNAcylation rhythm may contribute to the disruption in liver transcriptomic rhythm previously observed in DRF condition".

In our new site-specific analysis, we saw that some proteins actually exhibited inverted day-night O-GlcNAcylation patterns when mice were fed DRF instead of NRF (Fig 6E). This pattern was not obvious in the global O-GlcNAcylation analysis. It is possible that these proteins contribute to the previously observed DRF-induced phase shift of rhythmic genes (Damiola et al., 2000 G&D; Asher and Sassone-Corsi, 2015 Cell; Longo and Panda, 2016 Cell Metab; Manella et al., 2021 Nat Metab; Xin et al., 2021 iScience), but future functional experiments will be necessary to test this hypothesis. This is now discussed in the results and discussion of the revised manuscript (Lines 413-427, 502-511).

Finally, we also expanded our discussion (Lines 512-529) to provide a more balanced view. We clarify that in addition to nuclear O-GlcNAc proteome presented in this study, other mechanisms are also involved in regulating the rhythms of daily hepatic transcriptome in addition to O-GlcNAcylation of nuclear proteins. These additional mechanisms could include O-GlcNAcylation of proteins in other subcellular compartments. Briefly, O-GlcNAcylation of cytoplasmic proteins, e.g. Raptor (Xu et al., 2023 Mol Cell), has been shown to regulate gene expression. Other nutrient-sensitive signaling pathways or PTMs can also interact with nuclear and cytoplasmic protein O-GlcNAcylation. Our metabolomics data also showed that daily profiles of a number of metabolites were altered in response to DRF (Fig S9). For example, altered S-adenosylmethionine (SAM) can regulate gene expression through influencing histone

methylation (Wei et al., 2021 Cancer Res; Katada et al., 2010 Nat Struct Mol Biol; Aguilar-Arnal et al., 2015 Nat Struct Mol Biol). In summary, integration of nuclear and cytoplasmic protein O-GlcNAcylation, and other nutrient-sensitive pathways is required to better understand how meal timing regulates the daily transcriptome. Our goal for this study is to contribute to that understanding.

Secondly, Fig. 7B, Western blots of chemically labeled PTM proteins are not representative of the nuclear O-GlcNAcome. While the result implies the loss of rhythms among the most abundant nuclear PTM proteins, it does not rule out the effect of food entrainment on less abundant substrates such as CLOCK, BMAL1 and PER2. Immunoprecipitation of these transcription factors should benchmark Fig. 7B.

We agreed with the reviewer that results in Fig 7B (Now Fig 6B) are likely representative of the most abundant nuclear proteins, therefore only implies the loss of rhythms among the most abundant nuclear protein O-GlcNAcylation. We now recognized this limitation in Line 412-413.

Furthermore, we have now included TMT quantitative profiling of site-specific O-GlcNAcylation of liver proteins extracted from mice at two time-points, ZT11 (day) and ZT23 (night) to overcome this limitation (Lines 220-244). These mice were subjected to night-restricted feeding (NRF), same as mice used for label-free global O-GlcNAcylation analysis. Based on daily patterns of global protein O-GlcNAcylation in Western blot (Fig 1A, 1B) and label-free global O-GlcNAc proteomics (Fig 2D, 2E), which were performed at 6 time-points over the day-night cycle, we chose to analyze nuclear proteins from mouse liver collected at ZT11 and ZT23 because they represent a day and a night time-point that showed clear differences. In total, we detected 790 high-confidence O-GlcNAc peptides, belonging to 346 proteins. Over 40% of O-GlcNAcylated proteins identified by TMT site-specific analysis were also identified in our label-free global O-GlcNAc proteomics (Fig 2F). Multivariate PLS-DA analysis showed that O-GlcNAcylation sites quantified by TMT-MS exhibits day-night differences (Fig 2G). 125 O-GlcNAc sites on 101 proteins showed day-night O-GlcNAcylation differences (>20%-fold changes) (Fig 2H). Consistent with O-GlcNAcylation determined by Western blot (Fig 1A-B) and label-free global analysis (Fig 2D-E), over 60% of the 152 O-GlcNAc peptides quantified by TMT-MS display higher O-GlcNAcylation at ZT23 (nighttime) (Fig 2H and I). Notably, we also found sites that exhibit lower O-GlcNAcylation at daytime. We reasoned these sites potentially belong to less abundant nuclear proteins that are not representative in the profile shown in Western blot results (Fig 1A-B), highlighting the value of site-specific analysis, as suggested by the reviewer. Our new results provide further support for temporal regulation of site-specific O-GlcNAcylation over the day-night cycle.

To gain insight into whether site-specific O-GlcNAcylation is responsive to feeding time, we also performed TMT site-specific O-GlcNAc proteomics for nuclear proteins extracted from mice subjected to day-restricted feeding (DRF) and compared to that of NRF mice (Fig. 6D-F). Consistent with global nuclear protein O-GlcNAcylation detected by Western blot (Fig. 6B-C), DRF leads to reduced number of sites with day-night differences (from 128 to 100, i.e. 101 proteins to 74 proteins) with day-night differences in O-GlcNAcylation, as compared with that of NRF (Fig 6D). Notably, O-GlcNAcylation level in the majority of peptides is responsive to altered feeding time (Fig 6E), with some peptides showing inverted day-night patterns between

NRF and DRF. Similar to above, site-specific O-GlcNAcylation provides additional insights into the regulation of temporal O-GlcNAcylation. Our data support our conclusion that timing of food intake modulates daily O-GlcNAcylation rhythm in mouse liver (Lines 413-427, 502-511).

Thirdly, in Fig. 7B-D, are NRF data shown in Fig. 1?

Yes, the reviewer is correct. NRF data in Fig 7C and 7D (now Fig 6C and 6G) were the same as in Fig 1B and C. We have indicated this in the figure legend of Fig 7 (now Fig 6) (Lines 1368 and 1372-1373).

Fourthly, UDP-GlcNAc are compartmentalized in cells. Tissue UDP-GlcNAc is not representative of nuclear UDP-GlcNAc. This is a factor that may confound some of the interpretation, i.e. O-GlcNAcome is nuclear while UDP-GlcNAc is from bulk tissue.

We thank the reviewer for pointing this out. We added the discussion of this complexity in the discussion (Lines 518-519). Proteins could also be O-GlcNAcylated in the cytoplasm prior to nuclear import. Indeed, cytoplasmic O-GlcNAcylation has been shown to regulate nuclear shuttling of several transcription factors, including NF-κB p65 (James et al., 2002, Diabetes; Golks et al., 2007, EMBO J), Neuro D1 (Andrali et al., 2007, JBC), and CRTC2 (Al-Hakim et al., 2005, J. Cell Sci). We hope to analyze metabolomics and O-GlcNAc proteomics in nuclear- vs cytoplasmic- fractioned liver extract, with the goal to reveal how UDP-GlcNAc availability regulate O-GlcNAcylation in both compartments.

Lastly, metabolomics may include validated metabolites with circadian rhythms under NRF and DRF. For example, orinithine, SAH etc. Nevertheless, Fig. 7 does not affect the main conclusions of this study. It can be removed without altering the flow of this work.

We thank the reviewer for this suggestion. After much consideration, we decided to keep Fig S7 (now Fig S9) showing that diurnal profiles of several metabolites (including orinithine) were altered in response to feeding time to provide a broader picture of how metabolic input can impact the hepatic transcriptome. We also discussed in Lines 512-529 that O-GlcNAcylation is only one of the nutrient sensitive mechanisms that regulate gene expression. We suggested that the signals from cytoplasmic proteins could impact nuclear processes, other nutrient-sensitive signaling pathways, and other PTMs can also coordinate with O-GlcNAcylation to modulate gene expression.

Minor comments:
(1) Avoid natural and unnatural when describing TRFs. Muslims practice DRF for thousands of years.

We apologize for this oversight and we have edited the manuscript accordingly.

(2) Supplemental files are not accessible at this point. OGT and HCFC1 are typical O-GlcNAcylated proteins. In Fig. 2, omics profiles of OGT or HCFC1 can be shown as a quality control.

As the reviewer noted, we found many published O-GlcNAcylated proteins in our dataset, which was discussed in Lines 463-477. We identified O-GlcNAcylated HCFC1 and many NUP proteins in our label-free global analysis as well as newly included TMT site-specific O-GlcNAc proteome dataset. (File S2 and S5). We were able to detect OGT in our raw data for label-free global O-GlcNAc proteome, but OGT did not pass our statistical cutoff.

(3) TMT-phosphoproteomics has the advantage of deeper coverage, compared to label-free mass spectrometry. Fig. 3 would be strengthened by including a graph showing the number and some examples of newly identified rhythmic phosphorylation sites.

Thank you for reviewer's suggestion. We now added Fig S6D, a Venn diagram to show a comparison between the 1820 rhythmic phosphosites that we newly identified in this study to those previously identified in published datasets (File S7). All the phosphorylated peptides/proteins shown in Fig 3F are newly identified rhythmic phosphosites, except for SFPA. We also showed the common rhythmic phosphosites among our study and published studies in File S7.

(4) In Fig. 5E-F, CLOCK Ser427 is phosphorylated by GSK3beta, not the alpha form (Cell Cycle 8:24, 4138-4146)

We thank the reviewer for this correction and we have edited the manuscript and main figures accordingly.

Reviewer #3: In the manuscript # PBIOLOGY-D-24-01760R1, Liu et al. tried to explore the correlation between daily rhythms of O-GlcNAcylation and G-P interactive phosphorylation on transcription factors and gene expression rhythms of their targets in the liver. In brief, they observed daily oscillation of global nuclear protein O-GlcNAcylation in the liver of mice subjected to natural nighttime-restricted feeding (NRF). To infer the direct and indirect roles of rhythmic O-GlcNAc changes in regulating hepatic transcriptome, they conducted multi-omics analysis, including proteomics, O-GlcNAc proteomics, and phosphoproteomics. Their O-GlcNAc proteomic analysis suggest that 11.54% of 719 O-GlcNAcylated proteins are rhythmically O-GlcNAcylated over a 24-hour day/night cycle. To investigate proteome-wide G-P crosstalk, they performed parallel TMT phosphoproteomic analysis using the same liver samples. They then proposed that rhythmic O-GlcNAcylation might indirectly modulate thehepatic transcriptome by interacting with phosphorylation.

Overall it is an interesting study, as it suggests that mealtime may alter daily rhythm in nuclear protein O-GlcNAcylation to regulate hepatic gene expression. However, it suffers from shortcomings especially the omics data which have substantially compromised the novelty of this work and soundness of their conclusions.

Key points:

1. The authors performed O-GlcNAc chemoenzymatic labeling in combination with mass spectrometry proteomics to identify O-GlcNAcylated nuclear proteins in liver. However, no

information on O-GlcNAc peptides is available, regardless of O-GlcNAc sites. According to this reviewer, O-GlcNAc chemoenzymatic labeling coupled with mass spectrometry shall be able to provide O-GlcNAc peptide/site information (as can be seen from several papers: PMID: 28657654, PMID: 35254053, PMID: 34161081). However, such information is not provided. After a rough look at the list of O-GlcNAc proteins identified (S2 File), it is hard to believe that some proteins are O-GlcNAcyated, for example, several immunoglobulin proteins (e.g., A0A075B5P6 and A0A0A6YX66). It will be helpful if the authors can provide at least the glycopeptide information to show that such proteins are indeed O-GlcNAc modified. Moreover, none of the proteins shown in their list (S2 File) were validated experimentally. But in Figures 4-5, it appears that the authors tried to evaluate the interaction between the O-GlcNacylation and phosphorylation on modification sites of some proteins over a day/night cycle. Clearly, O-GlcNac sites on proteins are required to do such analysis.

We thank the reviewer for the suggestions.

After much optimization, we have now included TMT quantitative profiling of site-specific O-GlcNAcylation of liver proteins extracted from mice at two time-points, ZT11 (day) and ZT23 (night) (Lines 220-244) label-free global O-GlcNAcylation analysis. Based on daily patterns of global protein O-GlcNAcylation in Western blot (Fig 1A, 1B) and label-free global O-GlcNAc proteomics (Fig 2D, 2E), which were performed at 6 time-points over the day-night cycle, we chose to analyze nuclear proteins from mouse liver collected at ZT11 and ZT23 because they represent a day and a night time-point that showed clear differences. In total, we detected 790 high-confidence O-GlcNAc peptides, belonging to 346 proteins. Over 40% of O-GlcNAcylated proteins identified by TMT site-specific analysis were also identified in our label-free global O-GlcNAc proteomics (Fig 2F). Multivariate PLS-DA analysis showed that O-GlcNAcylation sites quantified by TMT-MS exhibits day-night differences (Fig 2G). 125 O-GlcNAc sites on 101 proteins showed day-night O-GlcNAcylation differences (>20%-fold changes) (Fig 2H). Consistent with O-GlcNAcylation determined by Western blot (Fig 1A-B) and label-free global analysis (Fig 2D-E), over 60% of the 152 O-GlcNAc peptides quantified by TMT-MS display higher O-GlcNAcylation at ZT23 (nighttime) (Fig 2H and I). Notably, we also found sites that exhibit lower O-GlcNAcylation at daytime. We reasoned these sites potentially belong to less abundant nuclear proteins that are not representative in the profile shown in Western blot results (Fig 1A-B), highlighting the value of site-specific analysis, as suggested by the reviewer. Our new results provide further support for temporal regulation of site-specific O-GlcNAcylation over the day-night cycle.

To gain insight into whether site-specific O-GlcNAcylation is responsive to feeding time, we also performed TMT site-specific O-GlcNAc proteomics for nuclear proteins extracted from mice subjected to day-restricted feeding (DRF) and compared to that of NRF mice (Fig. 6D-F). Consistent with global nuclear protein O-GlcNAcylation detected by Western blot (Fig. 6B-C), DRF leads to reduced number of sites with day-night differences (from 128 to 100, i.e. 101 proteins to 74 proteins) with day-night differences in O-GlcNAcylation, as compared with that of NRF (Fig 6D). Notably, O-GlcNAcylation level in the majority of peptides is responsive to altered feeding time (Fig 6E), with some peptides showing inverted day-night patterns between NRF and DRF. Similar to above, site-specific O-GlcNAcylation provides additional insights into

the regulation of temporal O-GlcNAcylation. Our data support our conclusion that timing of food intake modulates daily O-GlcNAcylation rhythm in mouse liver (Lines 413-427, 502-511).

We then used this new site-specific dataset to improve our analysis of O-GlcNAcylation-phosphorylation (G-P) crosstalk (Fig. 4), although we found that our site-specific analysis did not yield many O-GlcNAcylation sites with day-night differences that are in close proximity to rhythmic phosphorylation sites. But we found 109 pairs of O-GlcNAc sites and phosphosites in close proximity (Lines 332-341).

Finally, we performed functional analysis to validate O-GlcNAcylation at a phospho-degron of CLOCK (model shown in Fig 5B). Our chemoenzymatic labeling data showed reduced O-GlcNAcylation of CLOCK(S431A) as compared with CLOCK(WT), suggests S431 is indeed a O-GlcNAcylation site (Fig 5C and D) (Lines 355-366). We also performed transcriptional reporter assay in cell culture to show that O-GlcNAcylation at this motif regulates CLOCK transcriptional activity (Fig 5H) (Lines 386-404).

2. In page 9: "Our analysis was aimed at identifying O-GlcNAcylated proteins and quantifying global O-GlcNAcylation level over the day-night cycle, rather than identifying and quantifying O-GlcNAcylation at specific residues. The current technology to identify and quantify O-GlcNAc sites on proteins is not yet mature based on consultation with experts in the field, as the labile glycosidic bond of O-GlcNAcylation posed great challenge for mass spectrometry analysis [63]". According to this reviewer, at least several quantitative O-GlcNAc proteomics studies have been reported so far (e.g., PMID: 28657654, PMID: 35254053, PMID: 34161081). Thus, it seems to be possible to do such studies. Without sites, it might be problematic to draw solid conclusions between proteome-wide G-P crosstalk. Fig. 5C provides representative blots showing the daily rhythms of CLOCK O-GlcNAcylation. However, it is plausible that there is cross-talk at the modification sites on CLOCK and RBL2 (shown in other figures).

See response for comment #1.

3. The authors provided the information of phosphorylated peptides/sites (S5 File). However, key information (e.g., the localization score for the modification sites and of peptides) is missing. Thus, it is hard to judge the phosphoproteomics data quality. Assuming all sites provided are of high confidence, it appears the CLOCK protein has many phosphorylation peptides/sites, but these peptides appear to have different pvalues. In addition, some peptides have two phosphorylation sites (e.g., the peptide 'ETTAQSDASEIR' shows two sites S6 and S9). Maybe the two sites have similar changes? Site-specific phosphoproteomics is very mature nowadays (e.g., PMID: 34468274, PMID: 34857927). Quantitative phosphoproteomics at the site level will clearly help improve this study. This is especially important given that bioinformatic analysis throughout the manuscript and the conclusions (e.g., G-P cross-talk) are almost exclusively based on the omics data obtained.

Thank you for reviewer's suggestion to improve our study. We have added the score and m/z information to S6 File. We also noticed that some proteins have the same phosphosites on different peptides with different p values, while other peptides have multiple phosphorylation

sites. After consulting with Dr. Junfeng Ma, our collaborator with expertise on phosphoproteomics, we recognize that there is no ideal method to combine these information to show the quantification of each phosphosite. Therefore, we chose to show all phosphopeptides identified in the current studies to maximize transparency.

Minor points:

1. It is a bit strange the authors conducted only three biological repeats and observed batch effects on the relatively small sample sizes. Not sure whether there was big technical variability during sample processing and/or analysis. As we know, nowadays TMT can be readily used for the analysis of many samples with limited batch effects (e.g., https://pubmed.ncbi.nlm.nih.gov/32649874/)

We reanalyzed our label-free global O-GlcNAc proteomic data with 3 biological replicates (Fig 2), and the same livers were used for TMT phosphoproteome, which did not show much batch effects.

One TMT 18plex kit was used for three biological replicates for the phosphoproteomics, which improved data quality.

TMT MS was also used to perform the newly added site-specific O-GlcNAcylation analysis (n=4).

2. The term 'O-GlcNAcome' is awkward, which may be better described as 'O-GlcNAc proteome'.

We edited the manuscript as suggested by the reviewer.
