# Supplementary material for: O-GlcNAcylation of nuclear proteins in the mouse liver exhibit daily oscillations that are influenced by meal timing
Source: PLoS Biol. 2025 Sep 25;23(9):e3003400. doi: 10.1371/journal.pbio.3003400 (PMC12500093; doi:10.1371/journal.pbio.3003400)
Supplement: Attachment — Submitted filename: Response_250812.pdf [file pbio.3003400.s028.pdf]

Response Letter

We thank the editor and reviewers for their constructive comments and suggestions and for the opportunity to submit a revised manuscript. As suggested, we performed additional statistical analyses and provided additional data necessary to support the claims we made in the manuscript. We also provided clarifications when necessary. Below, we provide point-by-point responses that address the reviewers' concerns. Our responses are in black regular font, and the reviewers' comments are in *red italics font*. For the convenience of the reviewers, we are submitting a "marked-up" copy of the manuscript with key changes highlighted in red font.

Reviewer #1: The manuscript now contains substantial improvement to the original version and may serve as a valuable resource of O-GlcNAc proteome. However, throughout the manuscript, causal language is used for phenomena that are, at present, supported by correlational evidence (see below).

* *The title claiming O-GlcNAcylation "regulate hepatic gene expression," which imply causality, should be rephrased. Furthermore, given that no transcriptomic analysis was done, the mention of "hepatic transcriptomic rhythm" in the abstract and the discussion should be removed.*

As suggested by the reviewer, we changed the title to "Mealtime alters daily rhythm in O-GlcNAcylation to regulate nuclear proteins involved in hepatic gene expression". We also edited the manuscript to remove the mention of "hepatic transcriptomic rhythm" (Lines 40, 51-53, 140-142, 450-451, 462, 484, 516-517).

* *RAIN q value is inconsistently reported (some still report unadjusted p values), please provide it in all places where it is required. Given q<0.2 is quite permissive, the reader would benefit from clear indication of rhythms that has q<0.05.*

We have now provided RAIN q values in analyses where multiple testing was conducted for the -omics data (Fig. 2, 3, 4, S4, S9) (Lines 1355, 1381-1382). For Fig S7, please see figure legend for q values (Lines 1480-1482). In Fig. 5F (also shown in Fig. 5G), we observed daily changes in pCLOCK(S427) profile. Although the daily changes cannot be classified as rhythmic based on RAIN p value (p=0.066) and q value (q=0.496) from TMT phosphoproteomics analysis, we performed one-way ANOVA and determined that changes in pCLOCK(S427) are time-dependent (p=0.036). Both the RAIN q value and one-way ANOVA p-value for pCLOCK(S427) are now shown in the figure legend (Lines 1381-1382). In Fig. 5F, no q value is necessary for global CLOCK O-GlcNAcylation because that is the graphical representation of values measured by Western blots in Fig. 5E. Similarly, no q value is necessary for CLOCK protein level in Fig. 5G because that is the graphical representation of values measured by Western blots in Fig. 5E.

Similarly, we cannot calculate RAIN q values when multiple testing was not conducted for other targeted analysis, including in Western blots (Fig. 1B and 6C) and targeted metabolomics (Fig. 1C and 6G). That is the reason we only provided p values for those analyses.

Finally, we followed the reviewer's suggestion and now provided q values of individual peptides in S2 and S6 files to clearly indicate rhythms that has q<0.05.

**   The section title "Crosstalk between O-GlcNAcylation and phosphorylation (G-P) regulates hepatic protein function" should be rephrased, as it still contains speculative correlational data and causal claims such as line 308 "inhibit each other" should be replaced with correlational claims.*

As suggested by the reviewer, we changed the section title to "Identification of proteins that are modified by rhythmic O-GlcNAcylation and phosphorylation" (Lines 296-297). We have also tone down the claim of PTM crosstalk to be more correlative (Lines 304-309).

**   The Per2-luc assay indicates that OGT-dependent suppression of CLOCK transcriptional activity requires both S431 and S427. This is compatible with, but does not directly demonstrate, a model in which O-GlcNAc at S431 interferes with phosphorylation-dependent activation involving S427. In order to demonstrate O-GlcNAc at S431 inhibits phosphorylation, pS431 should be directly quantified in the presence/absence of OGT, using a specific phospho-antibody or mass spectrometry. Without it, the conclusion that it "demonstrate the crosstalk" or "modulates CLOCK transcriptional activity" is an overstatement, and should be rephrased.*

We agree with the reviewer that the results of the *Per2-luc* assay on its own (Fig. 5H) only demonstrates that both S431 and S427 regulate CLOCK transcriptional activity, but does not demonstrate G-P crosstalk at these 2 residues. But the rationale of our model depicting G-P crosstalk at CLOCK(S431) and functional consequence (shown in Fig 5B) is supported by (1) the presence of previously characterized G-P interplay motif in this region of CLOCK (Lines 349-352) and (2) prior published data by Spengler et al. (PMID: 19946213) reporting that S431 is the priming phosphorylation site for glycogen synthase kinase-3 (GSK3). Once CLOCK(S431) is phosphorylated, GSK3 phosphorylates S427, which promotes the degradation of CLOCK and affects its transcriptional activity.

Our TMT-phosphoproteomics data confirmed the results by Spengler et al. (PMID: 19946213) indicating that both S431 and S427 are phosphorylation sites. Using chemoenzymatic labeling of a CLOCK fragment (aa260-460), we provided evidence supporting that S431 is a O-GlcNAcylation site (Fig. 5C and D). Since both O-GlcNAcylation and phosphorylation modify the hydroxyl group on serine residues, S431 can only be modified by either O-GlcNAcylation or phosphorylation at a time. If phosphorylation of S431 is required for the phosphorylation of S427 by GSK3β, then O-GlcNAcylation of S431 will most likely block the substrate recognition of GSK3β. As a result, S427 is unlikely to be phosphorylated upon S431 O-GlcNAcylation. This model is depicted schematically in Fig 5B. Nonetheless, as suggested by the reviewer, the use of phospho-specific antibodies in the presence/absence of OGT can provide further support for this model. Unfortunately, these antibodies are not currently available. As recommended by the reviewer, we therefore rephrased our conclusion to avoid overstatement (Lines 405-409).

**   Line 441-442, "partially by influencing" implies causality, it should be replaced with e.g., "the change is associated with".*

We have edited the sentence to "the effect of timing of food intake on O-GlcNAcylation rhythm in mouse liver is associated with altered UDP-GlcNAc profile." (Lines 445-446).

**   Figure 4 requires more statistical tests. Spearman correlation needs to be controlled for*

multiple tests. The claim "in phase" "out of phase" also require appropriate test (for example, 4E is not visually apparent that the two lines are in phase). The statistical test that was used to demonstrate the day-night differences of figure 4I-L should be indicated.

We thank the reviewer for raising this point. After more careful review of recent -omics publications that analyzed phase shifting scenario between two conditions (PMID: 37365376, 40088888), we concluded that Spearman correlation is not an ideal way to describe "in phase" vs. "out of phase" rhythms. Therefore, we reanalyzed the data by comparing the phase differences of O-GlcNAcylation and phosphorylation on the same proteins using phase values calculated by RAIN. We defined the phase difference <= 2hr as phase locked ("in phase") and phase difference >= 8hr as phase shifted ("out of phase"), based on definitions used in previous publications (PMID: 37365376, 40088888) (Fig. 4A). The phosphorylation sites shown in Fig. 4C-D are "in phase" with phase difference as 0hr, while the phosphorylation sites shown in Fig. 4F-H have phase difference >= 8hr (Lines 304-309, 1347-1351).

For Fig. 4I-L, the presence of day-night differences was defined as having differences between day vs. night time-points larger than 20%. This criterion was previously used in published literatures on quantitative proteomics for O-GlcNAc sites (PMID: 26446791, 27213235) (Lines 1363-1364). We have now also described this criterion in the Method section Line 889-892.

**   Figure 5C is it nuclear extract or total lysate? Should be indicated, as nuclear extract should be used. 5C also lacks a loading control.*

In Fig. 5C, this experiment used a CLOCK(aa260-460) fragment to determine whether S431 residue is O-GlcNAcylated in cell culture. Give there are multiple O-GlcNAcylation sites on CLOCK, conducting this experiment using full-length CLOCK is more likely going to impede the observation of a reduction in O-GlcNAcylation signal in the S431A mutant variant. However, the downside of using CLOCK(aa260-460) fragment is that it may not recapitulate the localization of full-length protein. We therefore used total lysate to immunoprecipitate this fragment with α-FLAG and detected O-GlcNAcylation. Furthermore, since the goal of this experiment is solely to determine if S431 can be O-GlcNAcylated, we felt that the use of nuclear extract vs total lysate is not critical.

As for the loading control, it is standard practice to use the total level of targeted protein (inclusive of all isoforms) as a loading control to analyze protein PTM. Since we detected the level of CLOCK(aa260-460) O-GlcNAcylation, we normalized the O-GlcNAcylation level to the total CLOCK(aa260-460) to show that altered O-GlcNAcylation level was not due to the change of CLOCK(aa260-460) protein level in the S431A variant. Previous publications also used the same method for analyzing protein PTMs, and loading control using total protein stain or housekeeping proteins, ACTIN or HSP70, were not necessary and/or appropriate (PMID: 15980066, 35999195, 18593878, 39630861).

Reviewer #2: The authors have addressed all the concerns.

Reviewer #4: The paper by Liu demonstrates a periodic O-GlcNAcylation status in liver corresponding to day and night feeding. The paper has a substantial multi-Omics imprint with

provides a greater depth of understanding for this pleotropic post-translational modification.

1A: The authors should perform a western blot for a nuclear protein (lamins, histones, nuclear pore proteins) to demonstrate the load control. Moreover, the authors should show the cytoplasmic fraction to verify enrichment. Does the cytoplasmic fraction show time dependent changes in O-GlcNAc as well? A simple Rl2 (anti-O-GlcNAc) western blot would show this. The argument for focusing on only the nuclear fraction does not consider transcription factors moving into and out of the nucleus. Does OGT and OGA show periodic changes in nuclear or cytoplasmic localization?

We thank the reviewer for these suggestions. We used total nuclear protein detected by Coomassie staining as loading control because previous publications suggest that this is better for normalizing the Western blot signals compared to the use of a single housekeeping protein (PMID: 30914243, 36971113). Normalizing western blotting with total protein staining has also been recommended in Editorial articles in Journal of Biological Chemistry and American Journal of Physiology (PMID: 26657753; 29351459).

We confirmed quality of nuclear and cytoplasmic fractionation using Trypan blue and DAPI staining (as shown below). Trypan blue staining did not detect cytoplasmic debris (otherwise stained in blue), while DAPI DNA stain demonstrated fractionated samples were indeed nuclei. Nuclei were denoted in the images below by triangles, with colors corresponding to the same nuclei in Trypan blue and DAPI staining. Furthermore, when we used the nuclear fraction for proteomics analysis, the GO enrichment for cellular compartment showed that nucleus is the most significantly enriched (Fig. S3, S5 and S6).

Liver sample

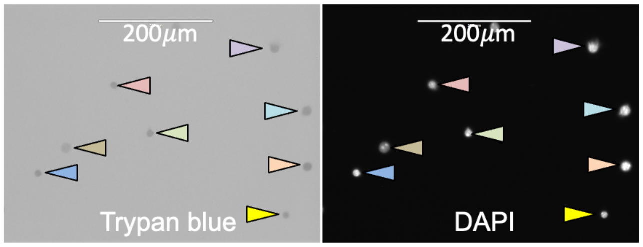

We agreed with the reviewer that O-GlcNAcylation detection in cytoplasmic fraction is certainly interesting and important to investigate. But given the already expansive scope of this current study, we felt that it is appropriate to reserve the analysis of cytoplasmic proteins for a future study. We did however discuss the potential roles of cytoplasmic protein O-GlcNAcylation in the discussion section, including translational regulation, nuclear shuttling of proteins, and signal transduction (Lines 518-521).

Finally, we agree with the reviewer that it is also interesting to investigate whether OGT and OGA show rhythmic nuclear vs cytoplasmic localization, since this could provide mechanistic insights into the regulation of rhythmic nuclear O-GlcNAcylation. In our previous paper (PMID: 34234137), we showed that nuclear OGT and OGA protein level display daily changes in *Drosophila* under natural feeding time. In the current study, our proteomic analysis showed nuclear OGT protein does not oscillate through a 24-hr period in NRF mouse liver (S3 file). It is possible that nucleocytoplasmic localization of OGT and OGA exhibit species- and/or

tissue-specificity. It is also possible that OGT and OGA activity are regulated at the post-translational level. These are all interesting questions that we hope to explore in future studies. We felt that including these experiments in this current study and further expanding the scope will detract from the main message of this study.

Figure 2: Its' unclear what is captured in the quantitation of the O-GlcNAc sites on peptides during the time points. Is it the O-GlcNAc is clock dependent or that the enrichment failed to capture the O-GlcNAcylated peptides. What would increase confidence is to orthogonally verify a protein that is changing during the light dark cycle. For example, EMSY O-GlcNAcylation has been validated before (Wang, Science Signaling, 2010). Ip EMSY and look for the change during the light dark cycle. That would increase rigor.

The quantitative PTM proteomic workflow used this study, where proteins were first digested and peptides with specific post-translational modification (PTM) were enriched for MS analysis, is a community standard practice for quantitative PTM proteomics (PMID: 38548019, 39531497, 34938717). For our TMT site-specific O-GlcNAc proteomics, the O-GlcNAc groups on proteins were first labeled by a biotin tag. This is followed by digestion of all the nuclear proteins into peptides by trypsin, TMT labeling of peptides, and enrichment of the peptides with biotin-O-GlcNAc modification using streptavidin affinity purification. After elution, the O-GlcNAc peptide were released and analyzed by mass spectrometry for O-GlcNAc site identification. We agree with the reviewer that validating some of these O-GlcNAcylation differences shown in our O-GlcNAc proteomics dataset could provide additional support, but this is often reserved for follow-up targeted studies. Nonetheless, in Fig. 5E and 5F, we did verify using IP (anti-CLOCK) in combination with chemoenzymatic O-GlcNAc labeling and Western Blotting that CLOCK protein, which was identified by our global O-GlcNAc proteomics analysis to exhibit daily changes, indeed showed daily rhythmicity in O-GlcNAcylation (RAIN, p=0.0040). We also highlighted that this profile detected by Western blot has similar trend to that detected by global O-GlcNAc proteomics (Fig S7A), supporting the validity of our global O-GlcNAc proteomics analysis using CLOCK as an example (Lines 363-367).

Overall, we felt that our data supporting the key message of this study - the presence of time-of-day differences in O-GlcNAcylation - is already supported by multiple sets of experiments in this study. First, in Fig. 1A and B, we showed that the global O-GlcNAcylation of nuclear proteins were rhythmic over a 24-hr period using chemoenzymatic labeling in combination with western blotting. Second, the label-free global O-GlcNAc proteomics identified 64 proteins with daily rhythmic O-GlcNAcylation (Fig 2A-E). Finally, the TMT site-specific O-GlcNAc proteomics revealed 125 O-GlcNAc sites on 101 proteins exhibit day-night difference (Fig 2F-I). Therefore, we feel that we provided strong evidence supporting our claim that O-GlcNAcylation of hepatic nuclear proteins exhibit time-of-day specific changes. We hope this study will encourage more in-depth, functional studies on specific protein targets identified in our datasets.

As for validating the O-GlcNAc sites with day-night difference identified in the TMT O-GlcNAc proteomic analysis, immunoprecipitating one target protein and detecting its O-GlcNAcylation over a 24 hr period (what we did for CLOCK mentioned above) would only show the profile of global O-GlcNAcylation on a given protein rather than a particular O-GlcNAc site. Unfortunately this does not achieve the goal of validating site-specific proteomics

data. Unlike site-specific phosphorylation, which can be detected using phosphospecific antibodies and Western blot, site-specific O-GlcNAcylation cannot be detected using similar reagents/method. We could use mass spectrometry after IP, but this would be using the same technique as what we had done in our proteomic analysis. It is also important to note that immunoprecipitating with a target protein antibody could also result in bias in the isoforms that are pulled down and may not accurately quantified isoforms that are modified by PTM. Overall, we feel that our PTM proteomics workflow follows best practices in the field (co-author Junfeng Ma is an expert and pioneer in O-GlcNAc proteomics methods development), but we recognize that future functional characterization will be necessary to fully validate specific O-GlcNAcylation sites and their functional consequences.

Figure 3: As above, rigor would be increased by performing a phosphorylation blot on one of the periodic phosphorylation sites.

We thank the reviewer for this excellent suggestion. Instead of focusing on one of the phosphorylation sites, we compared our entire phosphoproteomics dataset with published circadian phosphoproteomics datasets. We found that 80 of the rhythmic phosphorylation sites we identified were also reported in previous studies (PMID: 27818260; 27818261) (see Fig. S6D and S7 File).

Figure 6: As in Figure 2, showing the altered O-GlcNAcylation status via an Ip on an identified protein such as NCOR or GATAD2B would improve rigor.

Please see detailed response to earlier comment regarding validating site-specific O-GlcNAcylation.

Figure 6: Expression and localization data of OGT and OGA would help to identify if these proteins are different with the altered feeding schedule. The proteomics might even have the expression data?

We agree with the reviewer that it is certainly interesting to investigate whether OGT and OGA show rhythmic nuclear vs cytoplasmic localization, since this could provide mechanistic insights on the regulation of rhythmic nuclear O-GlcNAcylation. In our previous paper (PMID: 34234137), we showed that nuclear OGT and OGA protein level display daily changes in *Drosophila* under natural feeding time. In the current study, our proteomic analysis showed nuclear OGT protein does not oscillate through a 24-hr period in NRF mouse liver (S3 file). It is possible that nucleocytoplasmic localization of OGT and OGA exhibit species- and/or tissue-specificity. It is also possible that OGT and OGA activity are regulated at the post-translational level. These are all interesting questions that we hope to explore in future studies. The scope of this current study is already very expansive and would be difficult for us to include additional experiments to explore this aspect of the regulation.

In section headed: Daily G-P crosstalk on CLOCK, line 358 HEK cells are called "mouse HEK cells"

We apologize for this mistake. We have edited it as the reviewer suggested (Line 359).

The additional MS methods and data interpretation are good.
